# Pilot Study to Probe the Utility of Salivary Lactoferrin as a Stress Biomarker Relevant to Alzheimer’s Disease

**DOI:** 10.1002/alz.090856

**Published:** 2025-01-09

**Authors:** Valentina R. Garbarino, Hanzhou Wang, Chih‐Ko Yeh

**Affiliations:** ^1^ Glenn Biggs Institute for Alzheimer’s & Neurodegenerative Diseases, San Antonio, TX USA; ^2^ The University of Texas Health Sciences Center at San Antonio, San Antonio, TX USA; ^3^ The University of Texas Health Science Center at San Antonio, San Antonio, TX USA; ^4^ Audie L. Murphy division, South Texas Veterans Health Care System, San Antonio, TX USA

## Abstract

**Background:**

Approximately 6.7 million people in the US are diagnosed with an Alzheimer’s disease (AD), with greater incidence in women and minorities. Approximately 11 million family members provide uncompensated care to their family members with dementia, with more than 60% reporting high or very high levels of stress, a condition associated with increased risk for AD. Salivary lactoferrin, which plays a role in immune defense, may have utility as a biomarker for assessing stress with particular relevance to AD, allowing for earlier intervention. Decreases in salivary lactoferrin concentration are associated with stress in animal models, and reduced salivary lactoferrin levels have been associated with AD compared to normal cognition. Characterization of lactoferrin as a biomarker for stress is necessary to understand the mechanisms by which lactoferrin may mediate stress and contribute to AD pathogenesis.

**Methods:**

To probe the relationship between salivary lactoferrin and traditional indicators of stress, we utilized blood and saliva samples from females aged 49‐79 who serve as family caregivers for a parent/spouse with AD. Data was stratified by self‐reported ethnicity, Hispanic (N= 9) or non‐Hispanic (N= 9). Levels of salivary cortisol, IgA, and lactoferrin, and plasma C‐reactive protein were determined by enzyme‐linked immunosorbent assay.

**Results:**

Neither salivary cortisol (Mean Difference ± SEM, ‐0.010 ± 0.045; P=0.827) or lactoferrin levels normalized to salivary flow‐rate (59.77 ± 372.1; P= 0.875) were significantly different between groups. Lactoferrin concentration normalized to salivary flow rate was positively correlated with cortisol levels in non‐Hispanic females (Y = 6502*X – 440.0; R^2^= 0.5037; P= 0.0322). This correlation did not exist in the samples from Hispanic participants. Though there was a correlation between salivary cortisol and IgA levels in non‐Hispanic female participants (Y= 3.055e‐006*X +0.063; R^2^= 0.5627; P= 0.02), lactoferrin levels were not correlated with salivary IgA or plasma CRP.

**Conclusions:**

Salivary lactoferrin was positively correlated with cortisol levels in non‐Hispanic female caregivers. Future studies controlling for caregiver stress and potential resilience will be necessary to confirm these relationships and determine the mechanisms by which lactoferrin response to stress differs between Hispanic and non‐Hispanic populations, and may mediate risk of neurodegenerative disease.

Acknowledgements: P30AG044271 and TR002647.

